# Extrachromosomal Circular DNA: A New Target in Cancer

**DOI:** 10.3389/fonc.2022.814504

**Published:** 2022-04-14

**Authors:** Pan Wu, Yuhang Liu, Ruijia Zhou, Lingyun Liu, Hongli Zeng, Fang Xiong, Shanshan Zhang, Zhaojian Gong, Wenling Zhang, Can Guo, Fuyan Wang, Ming Zhou, Xuyu Zu, Zhaoyang Zeng, Yong Li, Guiyuan Li, He Huang, Wei Xiong

**Affiliations:** ^1^ NHC Key Laboratory of Carcinogenesis and Hunan Key Laboratory of Translational Radiation Oncology, Hunan Cancer Hospital and The Affiliated Cancer Hospital of Xiangya School of Medicine, Central South University, Changsha, China; ^2^ Key Laboratory of Carcinogenesis and Cancer Invasion of the Chinese Ministry of Education, Cancer Research Institute, Central South University, Changsha, China; ^3^ Hunan Key Laboratory of Nonresolving Inflammation and Cancer, Disease Genome Research Center, The Third Xiangya Hospital, Central South University, Changsha, China; ^4^ Cancer Research Institute, The First Affiliated Hospital, University of South China, Hengyang, China; ^5^ Department of Stomatology, Xiangya Hospital, Central South University, Changsha, China; ^6^ Department of Oral and Maxillofacial Surgery, The Second Xiangya Hospital, Central South University, Changsha, China; ^7^ Department of Medicine, Dan L Duncan Comprehensive Cancer Center, Baylor College of Medicine, Houston, TX, United States

**Keywords:** eccDNA, double-minute, cancer, heterogeneity, tumor biomarker

## Abstract

Genomic instability and amplification are intrinsically important traits determining the development and heterogeneity of tumors. The role of extrachromosomal circular DNA (eccDNA) in tumors has recently been highlighted. EccDNAs are unique genetic materials located off the chromosomal DNA. They have been detected in a variety of tumors. This review analyzes the mechanisms involved in the formation of eccDNAs and their genetic characteristics. In addition, the high-copy number and transcriptional levels of oncogenes located in eccDNA molecules contribute to the acceleration of tumor evolution and drug resistance and drive the development of genetic heterogeneity. Understanding the specific genomic forms of eccDNAs and characterizing their potential functions will provide new strategies for tumor therapy. Further research may yield new targets and molecular markers for the early diagnosis and treatment of human cancer.

## Introduction

Cancer is a major global public health problem with high morbidity and mortality rates and is a severe threat to human health receiving worldwide attention (1, 2).

Under normal physiological conditions, chromatin and related epigenetic mechanisms maintain specific gene expression patterns and cell homeostasis to adapt to various developmental and environmental conditions. However, abnormal genetic, environmental, or metabolic stimulation may lead to an excessively restrictive or loose epigenetic environment and chromatin aberrations, leading to cancer and other diseases (3). The occurrence and development of cancer depend on the coordination between complex and diverse molecular mechanisms. Abnormal gene expression is a hallmark of cancer (4, 5). The gene expression profile of cancer cells is the basis of their phenotype, which results from genomic aberrations, DNA methylation instability, and chromatin state aberrations (6). Oncogene behavior variation is one of the key cancer-driving factors. It includes changes in gene sequence, such as point mutations, chromosomal translocations, deletions, and insertions and changes in gene copy number, such as gene amplification and other gene activation mechanisms (7, 8), which facilitate large-scale DNA recombination (9). Genomic rearrangements drive cancer development through aberrations in chromosomal mechanisms, such as chromothripsis, which may lead to copy number changes, including the loss of tumor suppressor genes and an increase in the copy number of genes that promote malignant cell processes. Such recombination events involve the production of a special amplicon in the form of extrachromosomal circular DNA (eccDNA) (10–13), which leads to a chaotic genomic architecture that may result in oncogene copy number gain and tumor suppressor gene loss. Moreover, genome destruction has potential comprehensive carcinogenic consequences. For example, a double-minute (DM) chromosome containing the MYC proto-oncogene (*MYC*) is generated in the small-cell lung cancer cell line SCLC-21H through chromothripsis (14).

The eccDNAs currently studied in this paper are mainly large, copy number–amplified extrachromosomal circular DNA. EccDNAs are widely present in human tumor tissues and tumor cell lines, where they promote the amplification of oncogenes, driving tumor heterogeneity and drug resistance (15–19). This review mainly discusses the research progress of eccDNA in recent years, introduces the application value of eccDNA as a potential tumor marker and its influence in cancer evolution, and presents new challenges and opportunities for future anti-tumor therapy.

## The Characteristics of eccDNA

Human cells have 23 pairs of chromosomes. However, under certain conditions, some genes can be amplified in extrachromosomal DNA. There is a special class of circular DNA molecules that exist independently of the chromosomal genome, collectively referred to as eccDNA. They are separated or shed from the normal genome, free from the chromosomes, and are single- or double-stranded closed circular DNA structures (20, 21). They have also been found to be widespread in various eukaryotes, ranging in size, and are involved in specific ways in physiological or pathological processes such as aging, genome stability, cell communication, and tumorigenesis (22). Various types of eccDNA have been discovered as follows: (1) large, copy number-amplified eccDNA, commonly referred to in the text as ecDNA and DM, usually larger than 1 MB in size, including genes and noncoding DNA, widely found in various cancer types, driving tumor heterogeneity and drug resistance (23, 24). (2) small polydispersed circular DNA, ranging from a few hundred to several thousand base pairs, mainly derived from repetitive sequences organized in the genome, to initiate or enhance genomic instability (25–27). (3) microDNA, usually 100–400 base pairs, mainly derived from nonrepetitive genomic sequences with high GC content and exon density (28–31). and (4) telomeric circles, containing only telomeric repeats and involved in alternative lengthening of telomeres (32, 33). A more detailed classification and comparison of eccDNAs have been reviewed elsewhere (34, 35).

This review focuses primarily on the class 1 cancer-associated high-copy amplified eccDNAs, including DM and monomeric forms. In fact, paired DMs in tumor cells account for only 30% of these extrachromosomal elements (15). Such eccDNAs are acentric and atelomeric extrachromosomal elements, which are small fragments of chromosomes or chromatin particles that carry their own independent genetic information and regulatory regions. Usually, each eccDNA contains approximately 1 to 3 trillion base pairs or larger, is visible under light microscopy, which can be replicated in the early S phase (36). EccDNAs form supercoiled loops that function as miniature chromosomes. In 2019, Wu et al. used AmpliconArchitect to analyze WGS data and visualized the structural features of eccDNA through various imaging methods, which confirmed that eccDNA is circular (16). Xu et al. also used a new method combining multiple types of supporting genomic evidence, including graph search algorithm and chromium sequencing validation, to construct predicted circular structures representing DMs (37). Certain epigenetic modifications have also been detected in eccDNA molecules. Clarke et al. revealed the dynamic role of histone lysine methyltransferases and demethylases in regulating H3K4/9/27 methylation balance to control extrachromosomal amplification of the epidermal growth factor receptor (*EGFR*) oncogene (38). To date, few active histone modification markers and inhibitory histone markers on eccDNA molecules have been identified (16, 39).

## Mechanism of eccDNA Formation

Mechanisms involved in eccDNA formation have been proposed previously. For example, DMs may be related to genome rearrangements at the nucleotide sequence level in mammalian systems (40). DM generation may also be related to structural variation events (41, 42). Chromosomal studies have shown that chromosomes break and produce circular DMs in replication vesicles at stagnant bifurcations (43). Another hypothesis is that DMs may be caused by “looping out” chromosomal segments in the G1 or G2 phase (44). Large DMs may be formed by fusion of small extrachromosomal elements (45). The asymmetric separation of chromosomal fragments after chromosome breakage leads to the development and subsequent amplification of DMs. Large DMs can be formed directly without the need for smaller precursors, and DMs are stable in size (41, 46). DNA double-strand breaks (DSBs) trigger gene amplification in DMs through classical mechanisms such as unequal sister chromatid exchange, rolling cycle replication, break-induced replication, and folding back (47, 48).

**Figure 1:**
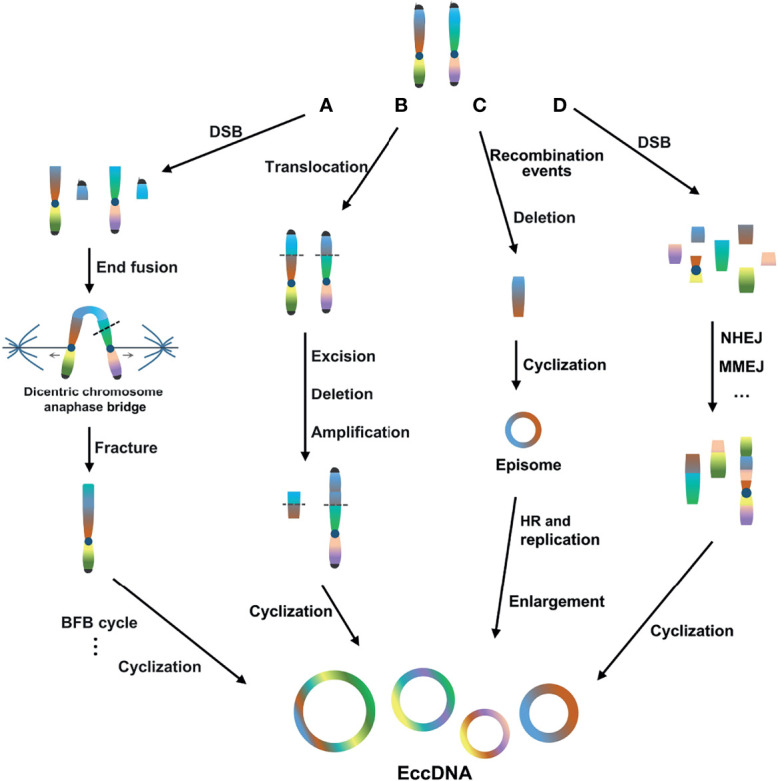
Four main models of eccDNA formation. **(A)** BFB cycle model. The BFB cycle begins with DSBs, where the ends of the broken chromosomes fuse to form dicentric chromosomes and anaphase bridges. Chromosomal bridge breakage at anaphase produces new ends lacking telomeres, triggering another round of amplification in the BFB cycle. **(B)** Translocation-excision-deletion-amplification model. Multiple DSB events trigger the formation of reciprocal translocations, and fragments near the translocation breakpoint can be amplified, excised, deleted, and circularized. **(C)** Episome model. The generation of episomes should be associated with the deletion of the chromosomal sequence corresponding to the recombination event. Episomes may be expanded by HR and duplication. **(D)** Chromothripsis model. DSBs occur on one or more chromosomes and then randomly recombine and circularize through DNA repair mechanisms such as NHEJ and/or MMEJ.

In general, the mechanism of eccDNA formation is mainly concentrated in four models. First, the break-fusion-bridge (BFB) cycle model is a common mechanism of gene amplification (49). The BFB cycle begins with DNA DSBs, where the ends of the broken chromosomes fuse to form dicentric chromosomes and anaphase bridges. Chromosomal bridge breakage at anaphase produces new ends lacking telomeres, triggering another round of amplification in the BFB cycle. The location and size of the breaks vary, resulting in the random generation of eccDNA (50, 51) (**Figure 1A**). Second, the translocation-excision-deletion-amplification model is characterized by multiple DSB events triggering the formation of reciprocal translocations, and fragments near the translocation breakpoint can be amplified, excised, and deleted, followed by circularization, leading to the generation of eccDNA (52). In this model, Van Roy et al. reported that non-direct co-amplification of MYC and ATBF1 is mediated by at least nine DSBs, resulting in the formation of mutually unbalanced t(8;16) translocations with excision, deletion, and amplification of sequences flanking the breakpoint (53). This mechanism has also been proposed to explain the co-amplification and expression of HMGIC and MDMD2 in precancerous pleomorphic adenoma carcinomas, accompanied by t(10;12)(p15;q15) translocation (54) (**Figure 1B**). Third, the episomal model, involving episome generation, should be associated with deletion of the chromosomal sequence corresponding to the recombination event. It may be expanded by homologous recombination and duplication, thus becoming visible as extrachromosomal DMs. For example, MYC-containing DMs in leukemia, neuroblastoma, and small-cell lung cancer cell lines are generated by excision and expansion (55, 56) (**Figure 1C**). Fourth, the chromothripsis model involves a catastrophic event in which DSBs occur on one or more chromosomes and then randomly recombine and circularize in an unnatural order and orientation through DNA repair mechanisms, such as non-homologous end joining (NHEJ) (57, 58) and/or microhomologous end joining (MMEJ) (59), to form eccDNA (60–62) (**Figure 1D**).

In recent years, research on the mechanism of eccDNA formation has tended to focus on the chromothripsis model (63–65). Vogt et al. found that the initial event in EGFR amplification was the formation of circular DNA molecules by the fusion of the two ends of a chromosome fragment through a microhomolog-based non-homologous end-joining mechanism. It was also determined that the amplicons of a particular tumor were derived from a single, established, circular DNA molecule, most likely produced by the excision of chromosomal fragments after duplication. These data extended the known role of this double-strand break repair pathway to the formation of DMs/eccDNA in tumors (24). Stephens et al. found that the small cell lung cancer cell line SCLC-21H contained a multitude of copy number changes in chromosome 8, showing a typical chromothripsis pattern. Chromosome 8 was broken into hundreds of fragments, many of which were stitched together to form a derivative chromosome 8, and 15 discrete segments that were amplified in large numbers were connected. One of these fragments contained the MYC oncogene, which, after a series of rearrangements and interweaving, finally formed a DM chromosome of approximately 1.1 Mb in size (14). Rausch et al., through whole-genome sequencing of medulloblastoma in patients with Li-Fraumeni syndrome, showed that in highly complex DNA rearrangements, inter- and intra-chromosomal amplified fragments physically connect to form a complex circular extrachromosomal structure (i.e. “DM” chromosome). The subsequent disruption of the telomeric fusion of two chromosomes by chromothripsis was considered to explain the origin of complex DMs (10, 66). The latest findings of Zhang Yi’s team also showed that eccDNA may be a circularization product of fragments generated by random genomic DNA fragmentation, which is related to apoptosis (67).

## eccDNA Is Detected in Multiple Tumor Types

EccDNA formation is a common event in cancers. Evidence of eccDNA in humans was demonstrated over half a century ago. As early as 1965, Cox et al. reported the discovery of eccDNAs in The Lancet, which were called DMs at the time. These were very small double chromatin bodies, which were abundant in some cells, observed during the metaphase in six tumor cases. The authors claimed that DMs were derived from chromosomes, not from bacterial contamination, and had no visible centromeres (68). Subsequently, eccDNA has been frequently found in many different tumor types and considered as an important indicator of abnormal cell behavior (69–73). For example, DM has been observed in patients with colorectal cancer (74). Amplification of the *c-Myc* oncogene in DM chromosomes has been reported in acute myeloid leukemia (AML) (75). Abnormal expansion of DMs is detected in approximately 1% of karyotypically abnormal AMLs and myelodysplastic syndromes (76). Loss of oncogenes in DM is significantly associated with decreased tumorigenicity (77). In 2017, a study in *Nature* reported that, based on integrated next-generation DNA sequencing and cytogenetic analysis of eccDNA, eccDNA is common in cancer, existing in nearly half of human cancers. Furthermore, its level varies according to the tumor type, but is very rare in normal tissue (78–80). EccDNA has been observed in nearly 40% of tumor cell lines and nearly 90% of patient-derived brain tumor models, most commonly in glioblastomas (81–84). In addition, it can be detected in varying degrees in neuroblastoma (85–87), adrenal carcinomas (88, 89), prostate cancers (90–93), breast cancers (94, 95), lung cancers (96), melanomas (97, 98), squamous cell carcinomas (99, 100), hematological malignancies (55) and other tumors.

The range and properties of eccDNA show specific differences between tumor and normal tissue, as well as before and after chemotherapeutic drug treatment. *MYC* and *EGFR* on eccDNA were amplified in 40% of cancer tissues, but no enrichment was found in normal tissues (101). Amplification of eccDNA occurs frequently in most cancer types, and the fact that eccDNA is more stable than linear DNA renders it a potentially prominent cancer biomarker. For example, abnormal amplification of DMs can be detected in various hematological malignancies, showing disease relevance (76, 102). It has been proposed that DMs in AML lead to reduced responsiveness to chemotherapy and, thus, a poor prognosis, which may be related to the number of DMs and their cytogenetic pattern (103). In fact, the results for AML patients with a complex karyotype containing DMs were discouraging, whereas those with a normal karyotype or only a single chromosomal aberration in addition to a large number of DMs seemed to fare better (104). In pediatric oncology, eccDNA, in the form of a DM chromosome containing the N-myc proto-oncogene, has become a definitive biomarker for clinical risk assessment in patients with neuroblastomas (105). Because the mechanism of early eccDNA formation in tumor evolution is still unclear, detection technologies have not been popularized, which increase the difficulty of early prediction and clinical application. Currently, there are few reports on other specific eccDNA biomarkers.

**Figure 2:**
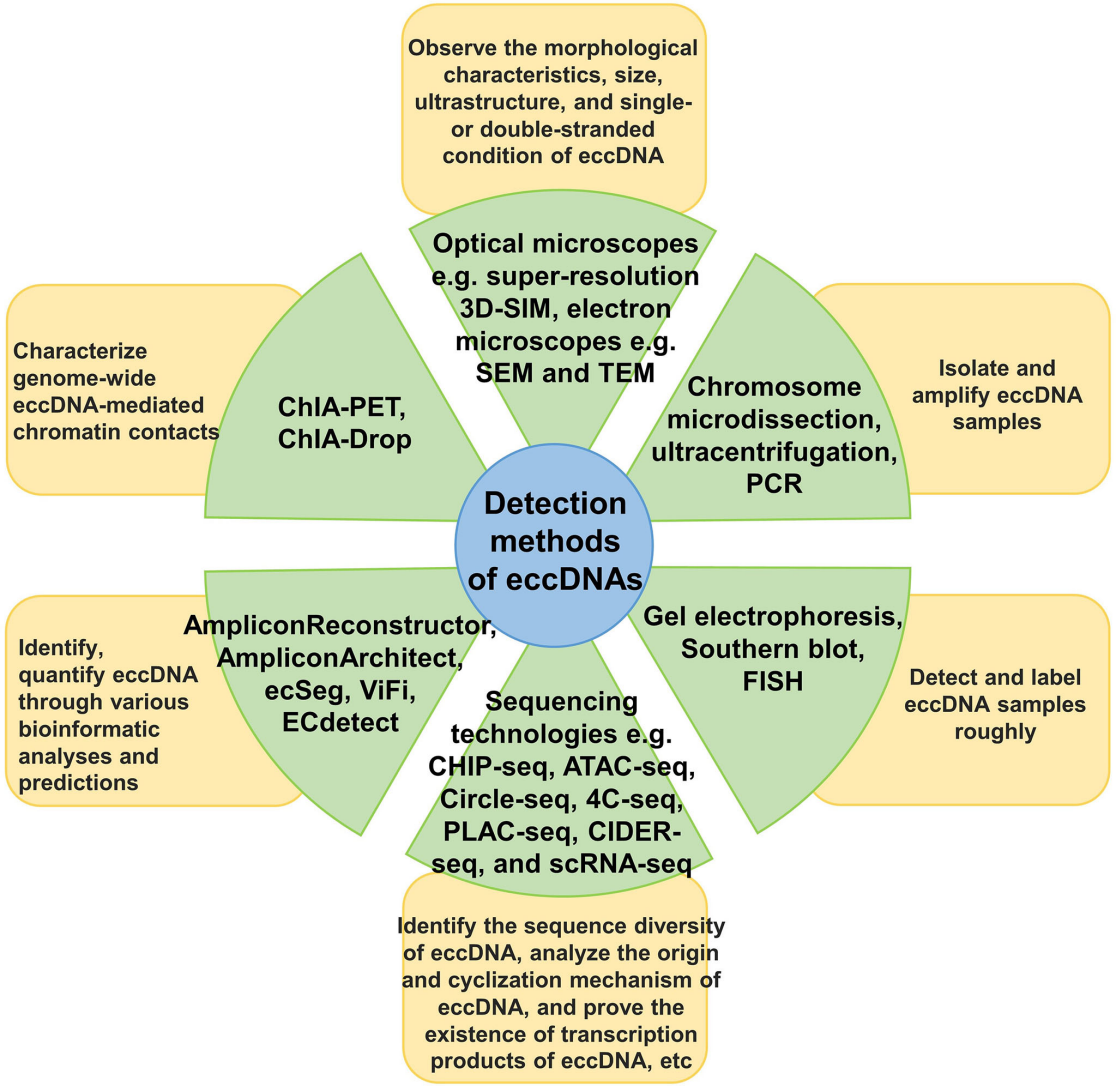
Detection methods for eccDNA. The main research and detection methods for eccDNA are classified and described, as well as the use of each type of method.

We have discussed the research and detection methods currently available for eccDNAs (**Figure 2**). For example, gel electrophoresis, Southern blotting, fluorescence *in situ* hybridization (FISH), polymerase chain reaction (PCR), and chromosome microdissection (106–108) can roughly identify and describe eccDNAs. The structure of eccDNA can be observed using light and electron microscopy (16, 23, 31). In addition, several high-throughput sequencing technologies, such as chromatin immunoprecipitation sequencing (ChIP-seq), assays for targeting accessible chromatin with high-throughput sequencing (ATAC-seq), circle-sequencing (Circle-Seq), circular chromosome conformation capture combined with high-throughput sequencing (4C-seq), proximity ligation-assisted ChIP-seq (PLAC-seq), circular DNA enrichment sequencing (CIDER-Seq), and single-cell RNA sequencing (scRNA-seq), have been developed and continuously optimized for analyzing the sequence diversity of eccDNA and the underlying mechanisms of its origin and circularization (27, 78, 109–111). Various visualization tools and bioinformatics analysis software, such as AmpliconArchitect, AmpliconReconstructor, ecSeg, ViFi, and ECdetect, have also promoted the understanding of eccDNAs (22, 35, 39, 112).

## Amplification and Expression Regulation of eccDNA ONCOGENES

The eccDNA molecule is an important structure for oncogene amplification *in vivo*. Oncogene amplification in eccDNAs, rather than in chromosomes, leads to higher copy numbers in tumors, often increasing the transcriptional abundance of oncogenes to the top 1% of the entire transcriptome (16, 56). Oncogenes, including the most common recurrent oncogenes, are highly concentrated on amplified eccDNA, thus leading to higher oncogene transcriptional levels. Oncogenes in tumor cells are amplified by extrachromosomal mechanisms to maintain cell-to-cell differences in oncogene copy numbers and transcription levels (80, 113, 114). EccDNA maximizes the proliferation and survival of tumor cells by increasing the expression level of oncogenes, thus, tumors become more aggressive (113).

**Figure 3:**
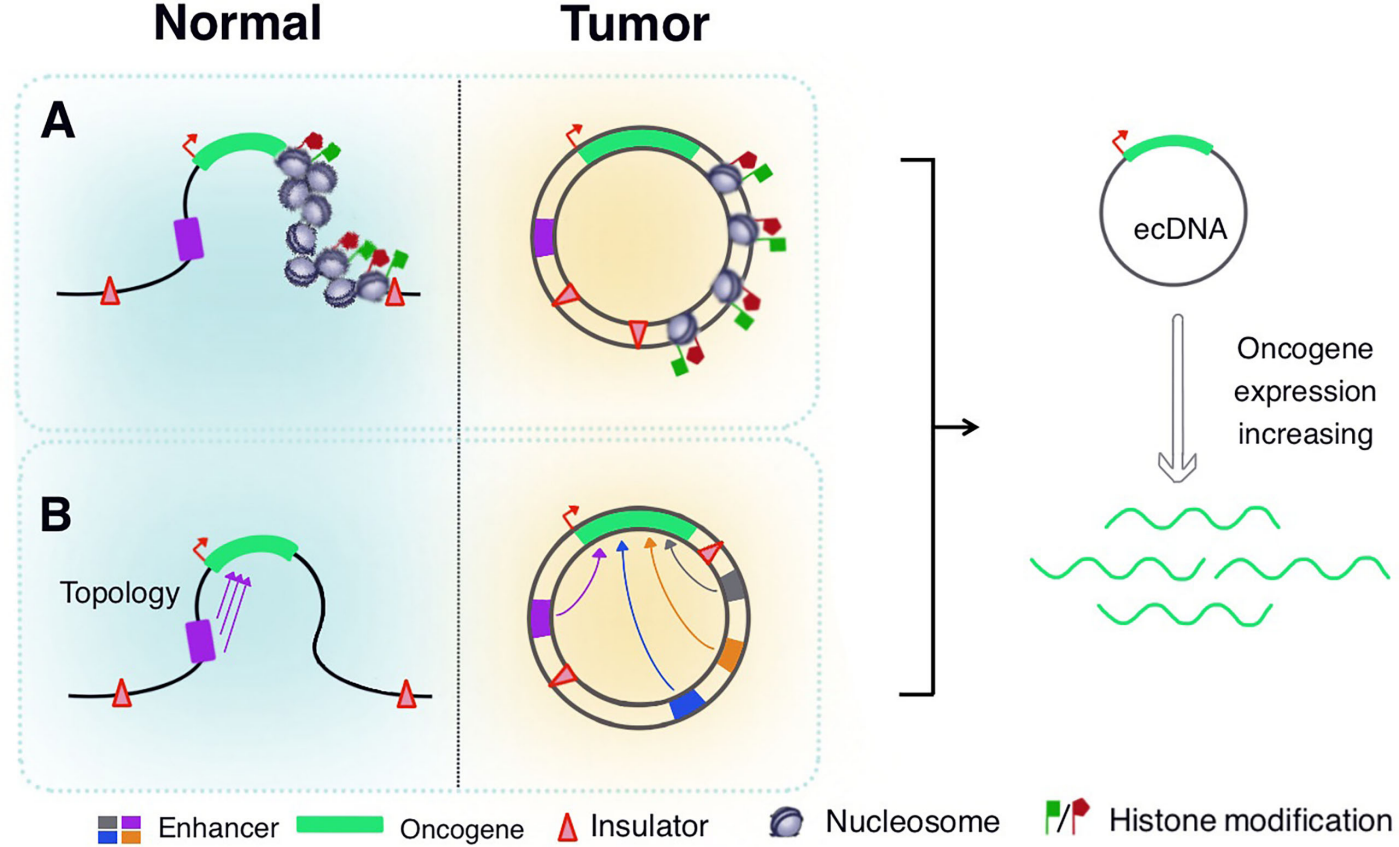
Regulation of eccDNA expression. **(A)** EccDNA is packaged into chromatin structures with complete domains, but lacks the typical higher-order chromosome compression, revealing significantly enhanced chromatin accessibility and histone modifications. **(B)** EccDNA chromatin forms a three-dimensional structure with topologically related domains, so that distant DNA elements become closer to achieve ultra-long-distance interactions. The oncogenes in eccDNA significantly enhance their self-regulatory activity through advanced amplification and circularization with adjacent enhancers.

A large number of oncogenes are expressed in eccDNA, which is attributed to its unique structure. Based on modern cancer genomics and epigenetic methods, eccDNAs have a highly open chromatin structure and support the distant regulation of DNA elements, non-coding DNA sequences, and other factors that regulate eccDNA expression (115) (**Figure 3**). Wu et al. used ATAC-seq, micrococcal nuclease digestion with deep sequencing, and ATAC-see chromatin visualization analysis to quantitatively evaluate eccDNA chromatin status. They have reported that although eccDNA is packaged into chromatin with a complete domain structure, it lacks typical higher-order chromosome compression and has a low degree of compaction. It exhibits significantly enhanced chromatin openness and active chromatin accessibility, allowing more access points and transcriptional sites to express their respective genetic information more quickly (16, 116). EccDNA chromatin also forms a 3D structure with topologically related domains. Simultaneously, the circular structure of eccDNA brings distant DNA elements closer, thereby building ultra-long-distance interactions and forming a new gene regulatory loop. The clustered regularly interspaced short palindromic repeats (CRISPR)/Cas9 system was used to inhibit *EGFR* transcription. The expression of distant genes in eccDNA is affected, which further suggests that the circular structure of eccDNA mediates DNA interactions over long distances (16). Aberrations in epigenome and transcriptome regulators play a pivotal role in carcinogenesis, as tumor cells often show abnormal promoter or enhancer activity at specific sites throughout the epigenome (117–119). Dysregulation of proto-oncogenes often involves mutations that bring transcription enhancers closer to these genes (120, 121). In November 2019, a study published in *Cell* reported that a large number of proto-oncogenes existed in the form of eccDNAs. However, a large number of non-coding DNA sequences were also detected, including enhancers and other regulatory elements. Additionally, amplification of extrachromosomal oncogenes was shown to be regulated by the non-coding genome (122). Morton et al. developed a computational method across five cancer types to identify the significant co-amplification characteristics of non-coding DNA outside the oncogene amplification range. In glioblastomas, *EGFR* and two related enhancers are in a 480 kb circular domain, co-amplification of *EGFR* with enhancers results in a dramatic change in the interaction pattern between the *EGFR* promoter and several adjacent cis-regulatory elements. CRISPRi screening revealed multiple newly acquired interactive enhancers, including two adjacent upstream enhancers, all of which had a strong positive effect on the activity of *EGFR*-amplified tumor cells. These data demonstrate that oncogenes in tumor cells significantly enhance their self-regulatory activity through advanced amplification and circularization (122). Subsequently, a study by Koche et al. showed the amplification and circularization of many neuroblastoma-related genes in eccDNA, such as *MYCN*, Jun proto-oncogene (*JUN*), MDM2 proto-oncogene (*MDM2*), SRY-box transcription factor 11 (*SOX11*), and T-cell acute lymphocytic leukemia 2 (*TAL2*). This finding is consistent with previous results showing that oncogenes are circularized and co-amplified with neighboring enhancers. Extrachromosomal circularization is a potential driving force for high-level local genome amplification, and the article also suggested that the circle length is a related factor affecting copy number (123). In 2021, Zhu et al. reported that eccDNAs could bind to RNAPII and mediate extensive chromosomal contact. The contact sites of eccDNA and its chromosomal targets are mainly localized in non-coding regions with high H3K27ac levels. This suggests that the enhancer signal gathered at the eccDNA-chromatin contact sites, and the chromatin contacts activated the chromosome enhancer to further increase the transcription of oncogenes, showing specific super-enhancer modifications. eccDNA acts as a mobile enhancer for the transcriptional activation of chromosomal genes (39, 124, 125). These features greatly expand the dynamic plasticity of oncogene expression regulation in space, promote cancer pathogenesis and the expression of abundant growth-promoting oncogenes in tumor cells, and facilitate quicker responses to changing environments and potential threats.

## EccDNA Drives Tumor Evolution and Genetic Heterogeneity

**Figure 4:**
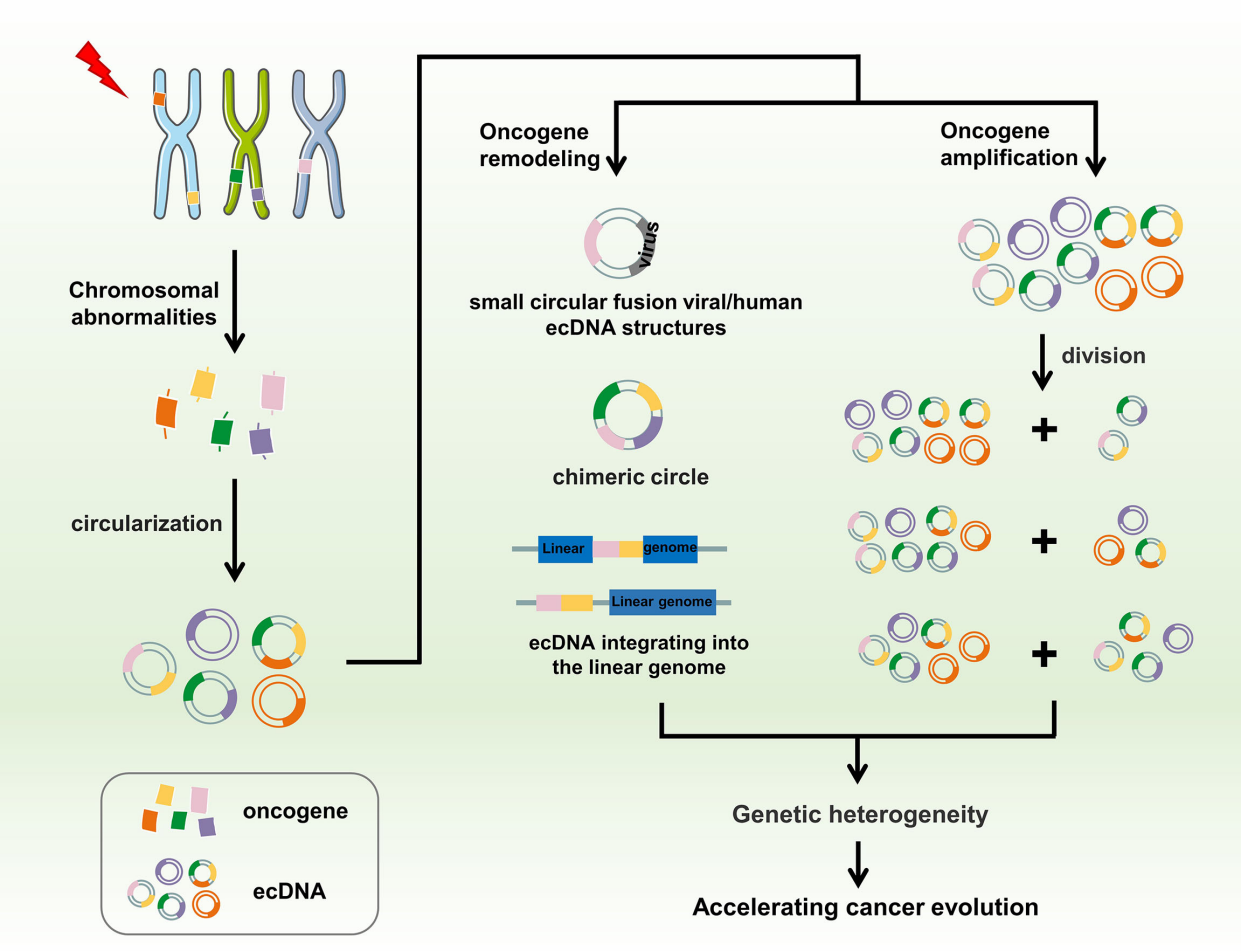
EccDNA accelerates cancer evolution. Chromothripsis, genomic rearrangement, or other possible chromosomal abnormalities may cause DNA fragments to circularize to form eccDNA. EccDNA promotes oncogene remodeling through fusion with human viruses or chimeric circularization and reintegration of eccDNA into the linear genome, whereas amplification of oncogenes on eccDNA instead of chromosomes leads to higher copy numbers of these oncogenes in tumors. Moreover, eccDNA is randomly distributed to progeny cells during mitosis owing to the lack of centromeres. Ultimately, these processes promote genetic heterogeneity, further accelerating cancer evolution.

Tumor complexity has been attributed to tumor heterogeneity at the molecular, genomic, and epigenetic levels, in addition to various environmental pressures (74, 78, 79, 86, 122). The existence of eccDNA is an important factor driving genetic heterogeneity in tumors. Owing to the lack of centromeres, eccDNAs are gradually lost during mitosis (126, 127). They do not follow Mendel’s law of inheritance and are randomly distributed to daughter cells. Consequently, one of the daughter cells may have multiple oncogene copies of eccDNA during each division, thereby gaining proliferation advantage (80, 128). Ploidy abnormalities and gene amplification are common features of malignant cells. This differential distribution also determines how eccDNA functions in cells, significantly differing from traditional chromosomal DNA. Amplification of eccDNA enhances genomic diversity and promotes heterogeneity. It also rapidly increases and is sustained longer in tumors, a distinct genetic process that allows cells to undergo more rapid evolution (84, 129). Studies have verified the amplification of 34 genes encoded in eccDNAs, including *EGFR*, *MYC*, cyclin-dependent kinase 4, MET proto-oncogene, *MDM2*, and platelet-derived growth factor receptor alpha in the interphase and mid-term through FISH in glioblastoma samples. In all FISH experiments in interphase, the number of fluorescent signals in each cell nucleus varied greatly, ranging from 2 to 100. This obvious heterogeneity indicates that the number of target gene DNA copies in each cell is different, which leads to differences in eccDNA amplification (84). *EGFR* is the most commonly amplified gene in gliomas, accounting for approximately 40% of all amplified cases (130). *EGFR* amplification has significant specificity for glioblastomas (24, 131). However, there is no clear explanation for the relationship between eccDNA structure and oncogene amplification. The structure and behavior of eccDNAs also varies among different types of cancer (114, 132). EccDNA is not only the driving force of eukaryotic genome instability but is also a product of programmed genome rearrangement (70, 133). Nguyen et al. have reported that genome rearrangement is associated with virus integration in eccDNA, thus forming small circular fusion viral/human eccDNA structures, contributing to the pathogenesis of HPV-related cervical cancer, which may be due to the indiscriminate transcription of proximal genomic elements in the circular structure. The eccDNA provides a complementary mechanism for the pathogenesis of certain virus-related cancers (134). Deshpande et al. have also confirmed the fusion of human virus-extrachromosomal DNA using AmpliconArchitect. EccDNA may play an important role in creating complex rearrangements and focal oncogene amplification, thereby driving the growth of multiple cancer types (135). A common manifestation of plasmacytoma variant translocation 1 (*PVT1*) locus amplification is the formation of DMs, which then participate in DM amplification or form new fusion genes, resulting in high *PVT1* expression in tumors and tumorigenicity (136, 137). In AMLs, the new fusion RNAs, *PVT1*- non-smc element 2 (*NSMCE*2), and coiled-coil domain-containing protein 26-*NSMCE2*, have been associated with DM derived from chromosome 8, showing 8q24 amplification, indicating that the carcinogenic effect of the amplified fragment of chromosome 8q24 is related to the DM chromosome (138, 139). A study published in *Nature Genetics* found that eccDNA drives oncogenic genome remodeling in neuroblastomas. By combining genomic and transcriptomic approaches, the authors unexpectedly discovered that eccDNA is the main source of somatic genome rearrangement, which promotes oncogene remodeling through chimeric circularization and circular DNA reintegration into the linear genome. Circle-derived rearrangements cause carcinogenic lesions and abnormal expression of tumor suppressors and proto-oncogenes (123). The presence of eccDNA is important for cancer cell phenotypes. The number of eccDNA molecules in cancer cells, number of amplified genes in eccDNA, or increased number of therapeutic resistance genes may deteriorate the phenotype of cancer cells, leading to continued proliferation, expression of differentiation markers, or reduction of apoptotic cell death, and promoting adaptive evolution of tumor cells. Thus, eccDNA is associated with multiple mutations and mutagenic processes related to rapid disease progression and short survival (140, 141). EccDNA is an important genomic feature of tumors, as it changes the distribution and rearrangement modes of cancer-related oncogenes, enables rapid heterogeneity of tumor cells, promotes malignant behavior, and accelerates the evolution of human cancer (**Figure 4**).

## EccDNA Is Associated With Tumor Drug Resistance and Poor Prognosis

EccDNAs contribute to tumor drug resistance through multiple mechanisms. In the last century, it was discovered that 17% of the bone marrow cells from a patient with AML had DM chromosomes, which were resistant to cytarabine and daunorubicin hydrochloride. The patient died within 3 weeks of symptom onset. This prompted scientists to examine whether there is an association between eccDNA and tumor resistance or disease progression (142, 143). The amplification of oncogenes, such as *C-MYC* and mixed-lineage leukemia, on eccDNA in leukemia is usually associated with shorter patient survival time and poor chemotherapy effects (141). Subsequent studies on glioblastomas arrived at a similar conclusion that eccDNA heterogeneity allows tumor cells to survive chemotherapy or radiation (84). In methotrexate treatment, the development of drug resistance is associated with unstable amplification of the DM-related dihydrofolate reductase (*DHFR*) gene (144, 145). Additionally, *DHFR* in eccDNAs is greatly amplified following methotrexate treatment. *DHFR* expression overcomes the inhibitory effect of methotrexate on folate metabolism and maintains DNA anabolic activity in tumor cells, leading to drug resistance (146, 147). Nathanson et al. observed, through single-cell analysis of models and clinical samples from patients with glioblastomas treated with EGFR tyrosine kinase inhibitors (TKIs), that tumor cells reversibly upregulated or inhibited the expression of extrachromosomal DNA EGFRvIII, resulting in a different cell phenotype with the best growth trade-off. The loss of extrachromosomal DNA EGFRvIII is a common and clinically relevant EGFR TKI resistance mechanism in glioblastoma multiforme (148, 149). Xu et al. have reported that DM evolution is associated with drug resistance in brain tumors. Among the DMs identified in pediatric patients, the abundance of *EGFR* in different samples varied and was also altered in diagnostic and recurrence samples. All DM copies carrying *EGFR* in patients who relapsed after erlotinib treatment contained secondary somatic deletions. However, the frequency of somatic mutations at the time of diagnosis was very low, indicating potential resistance to EGFR inhibitors. The authors observed a similar pattern involving the longitudinal copy number shift of DMs in another four pairs (diagnosed/relapsed) of adult glioblastomas. Although the same oncogenes were amplified during diagnosis and recurrence, they were amplified on different DMs (37). Koduru et al. reported that patients with breast cancer developed secondary AML within 18 months of using trastuzumab, pertuzumab, docetaxel, carboplatin, and other drugs to assist radiotherapy, which is probably caused by *MYC* and *KMT2A* amplification as DMs. Deletion and mutational inactivation of tumor protein p53 amplifies various genes at different levels in different DMs, causing genetic abnormalities called “double-minute heterogeneity” in leukemic cells (150). Recurrent tumors are often accompanied by complex genomic changes, extensive mutations, and copy number heterogeneity on DMs (151). Many other oncogenes have been identified on extrachromosomal DNA, which quickly respond to targeted drug therapy. The final existence and quantity of different eccDNAs depend on how they promote tumor growth or develop drug resistance. The mechanism of eccDNA involved in drug resistance against targeted cancer treatments is complex and diverse (30, 82, 152). Amplification-linked extrachromosomal mutations facilitate effective adaptation of tumor cells to various environmental conditions, including environmental changes caused by anti-cancer treatments (82). Tumors amplified with eccDNA appear aggressive and have a significantly poorer prognosis. Statistical analysis of whole genome sequencing from 3212 patients found that cancers with eccDNA amplification were associated with invasive characteristics. Tumors containing eccDNA amplification showed greater lymph node spread at initial diagnosis, and the patients showed a lower 5-year survival rate than those without eccDNA amplification (114). This suggests that eccDNA affects cell growth and treatment response by promoting rapid changes in the number or structure of oncogenes in tumors, and plays a key role in specific tumor drug tolerance and poor prognosis.

## Challenges and Opportunities

Currently, the existence and importance of eccDNA is undisputed. EccDNA in human cancer cells differs from that in normal chromosomal DNA. It provides new insights into the 3D structure of the chaotic cancer genome and epigenome, and the aggressive nature of specific tumor cells. This review summarizes the progress on eccDNA research over the recent years and raises additional questions and challenges. Therefore, future research on eccDNA may be performed along the following lines. For example, the specific process underlying the eccDNA-mediated regulation of mRNA transcription and whether additional epigenetic modifications play a role in eccDNA biology remains unclear. Furthermore, the biogenesis and circularization mechanism of eccDNA has not yet been clarified. EccDNA and chromosomal DNA differ greatly in critical aspects, such as location, topology, and biology. Therefore, the development of new research tools, such as the optimization of high-throughput sequencing analysis tools, is urgently required to further explore the amplification of oncogenes *via* eccDNA and unravel the role of eccDNA in cancer pathogenesis (153). Previous studies have focused on the functions of oncogenes, but the regulatory elements that control oncogene expression have been ignored. Future studies should address how switches that activate these oncogenes can be turned off. EccDNA is a carrier for oncogene amplification, with a complex structure, high frequency in tumors, and powerful ability to be re-inserted into the genome. Perhaps, it is possible to develop eccDNA into functional carriers in future therapeutic approaches. Moreover, these eccDNAs may play a key role in tumor evolution, resistance to specific treatments or drugs, and poor prognosis, all of which currently impose significant limitations on the development of new anti-cancer therapies. A better understanding of the degree of heterogeneity of crucial cancer genes and mutation processes in tumors, and their dynamic changes over time is essential (154–157).

Nonetheless, the large number of eccDNAs expressing oncogenes is vital for promoting rapid tumor growth and may be used clinically for cancer research, treatment, diagnosis, and prognosis. Tumor tissues release eccDNA into the circulatory system, which can be used for cancer fluid biopsies to monitor disease progression (29, 158, 159). Eliminating eccDNA from cancer cells to interfere with tumor growth, treat drug resistance, and prevent tumor recurrence is a potential strategy for cancer treatment. In some cases, non-cytotoxic doses of hydroxyurea may reduce the number of DMs and their mid-term spread in cancer cells in patients with advanced ovarian cancer (160). Hydroxyurea treatment accelerates the loss of extrachromosomally amplified drug resistance genes in tumor cells, accompanied by a consequent increase in drug sensitivity (161). In ovarian cancer cells, the anti-cancer drug gemcitabine effectively eliminates eccDNA, thereby slowing the growth and proliferation of tumor cells (162). Sanchez et al. reported that graded ionizing radiation accelerated the loss of the amplified multidrug resistance gene *MDR1* carried by extrachromosomal DNA in tumor cells, improving the treatment response for some cancers (163). In colon cancer cells, silencing *BRCA1* inhibits homologous recombination, reducing the extrachromosomal amplification of methotrexate-resistant cells and improving the chemotherapeutic effect against tumors (164). TKIs help eliminate mutated eccDNA in glioblastomas and low-grade gliomas (165).

In future, eccDNA may be used as a new target for cancer-targeted therapy. Alternatively, it may be combined with traditional radiotherapy and chemotherapy or with classical anti-cancer drugs to boost the effectiveness and synergy in tumor therapy. The unique structure and function of eccDNA in tumorigenesis makes it a potential new target for drug development (162, 166). EccDNA heterogeneity levels within and between tumors may be studied to tailor personalized treatment regimens for individual patients with tumors (108). The eccDNA structure contains 20–50 enhancers and other regulatory elements. Researchers are trying to find a class of therapeutic drugs that inhibit these regulatory components, providing a new way to inhibit oncogene function (122). The differential expression and prognostic relevance of eccDNA in cancer may be foreseeable in future through more rigorous experimental analysis and clinical examinations, such as immunohistochemical analysis of tumor tissue paraffin sections and examination of body fluid and blood specimens (167, 168). An interesting possibility is that eccDNA, because it is not protected by chromatin, may act as an endogenous antigen that activates autoimmune pathways. It is released into the cytoplasm during mitosis and activates the cyclic guanosine monophosphate-adenosine monophosphate synthase pathway, thereby stimulating the innate immune response (101). Studies have also reported that eccDNA has a strong ability to stimulate innate immune responses, which depend on the intracellular Sting signaling pathway (67). Furthermore, eccDNA has the potential to be useful in anti-tumor vaccines. Thus, emerging therapies targeting eccDNA may revolutionize anti-tumor therapy following chemoradiotherapy, targeted therapy, and immunotherapy.

## Conclusions

It has been discovered that abnormal oncogene amplification is abundant in extrachromosomal DNA. These eccDNA molecules have circular structures, and chromothripsis and genome rearrangement may greatly influence their formation. EccDNA is ubiquitous in various human cancers. EccDNA enables high copy numbers and high expression of oncogenes, thereby promoting tumorigenesis and adaptive evolution. It is randomly distributed during cell division, drives oncogene remodeling, and changes the expression patterns of oncogenes. EccDNA is an important driving force for drug resistance and is associated with poor prognosis as well as the development of genetic heterogeneity. Understanding the structure and function of eccDNA will enhance our understanding of tumor pathogenesis and provide insights for new cancer treatment strategies and clinical applications. In future, a more in-depth exploration of eccDNA is required to reveal the regularity and implications of this exciting phenomenon.

## Author Contributions

PW, YHL, RZ, LL, HZ, and FX collected the related paper and finished the manuscript and figures. SZ, ZG, and WZ gave constructive guidance and made critical revisions. CG, FW, MZ, XZ, ZZ, YL, GL, HH, and WX participated in the design of this review. All authors read and approved the final manuscript.

## Funding

This work was supported by the National Natural Science Foundation of China (81672683), the Natural Science Foundation of Hunan Province (2018SK21211), and the 111 Project (111-2-12).

## Conflict of Interest

The authors declare that the research was conducted in the absence of any commercial or financial relationships that could be construed as a potential conflict of interest.

## Publisher’s Note

All claims expressed in this article are solely those of the authors and do not necessarily represent those of their affiliated organizations, or those of the publisher, the editors and the reviewers. Any product that may be evaluated in this article, or claim that may be made by its manufacturer, is not guaranteed or endorsed by the publisher.

## Abbreviations

eccDNA, extrachromosomal circular DNA; DM, double-minute/double minute; BFB, break fusion bridge; DSB, double strand breaks; HR, homologous recombination; NHEJ, non-homologous end joining; MMEJ, microhomologous end joining; FISH, fluorescence *in situ* hybridization; PCR, polymerase chain reaction; 3D-SIM, three-dimensional structured illumination microscopy; SEM, scanning electron microscope; TEM, transmission electron microscope; CHIP-seq, chromatin immunoprecipitation sequencing; Circle-Seq, circle-sequencing; PLAC-seq, proximity ligation-assisted ChIP-seq; 4C-seq, circular chromosome conformation capture combined with high-throughput sequencing; ATAC-seq, assay for transposase accessible chromatin using sequencing; CIDER-Seq, circular DNA enrichment sequencing; scRNA-seq, single-cell RNA sequencing; ChIA-PET, chromatin interaction analysis by paired-end tag sequencing; ChIA-Drop, chromatin interaction analysis with droplet sequencing; CRISPR, clustered regularly interspaced short palindromic repeats; TKIs, tyrosine kinase inhibitors; EGFR, epidermal growth factor receptor; MDM2, MDM2 proto-oncogene; MYC, MYC proto-oncogene; PVT1, plasmacytoma variant translocation 1; AML, acute myeloid leukemia; DHFR, dihydrofolate reductase gene.
